# PLD1 overexpression promotes invasion and migration and function as a risk factor for Chinese glioma patients

**DOI:** 10.18632/oncotarget.18961

**Published:** 2017-07-05

**Authors:** Wenjun Tang, Richu Liang, Yonghong Duan, Qiaoling Shi, Xiaofei Liu, Yongshi Liao

**Affiliations:** ^1^ Department of Neurosurgery, The Second Hospital Affiliated to University of South China, Hengyang, Hunan, 421000 China

**Keywords:** PLD1, glioma, prognosis, invasion, migration

## Abstract

Glioma is a lethal disease with few effective therapeutic options. Recently, insights into cancer biology had suggested that abnormal lipid metabolism was a risk factor for various human malignancies, including glioma. As a key enzyme implicated in lipid metabolism, PLD1 was overexpression in multiple human cancers, and it was stated to be responsible for aggressive phenotypes, such as angiogenesis and chemoresistance. However, there was still much to know about its expression and function in glioma. In the present study, we showed that PLD1 was overexpression in clinical samples of glioma. In addition, the correlation assay revealed that PLD1 overexpression was correlated with poor differentiation (*p* = 0.04), and it was responsible for a poor prognosis for the patients (*p* = 0.009). Furthermore, we showed in COX regression assay that PLD1 was a risk factor for glioma (*p* = 0.018, HR = 0.461, 95% CI = 0.243–0.887). Consistently, we found that PLD1 was overexpression in glioma cell lines, and it could facilitate the proliferation and migration. Taken together, our study suggested that PLD1 was pro-tumoral in glioma, and that further studies were urgently needed so as to define whether it was a novel therapeutic target for the disease.

## INTRODUCTION

Glioma is one of the most common primary cancers of brain in phenotype, and it was the first cause of brain cancer-related deaths among all brain tumors [1, 2]. Recently, lots had been been obtained toward the molecular and cellular biology of the lethal disease during the past decade, but long-term survival for the patients remains poor, especially for those diagnosed with glioblastoma, which was characterized by high chemo-and radio-resistance [3]. The tumorigenesis of glioma is multifactorial with the involvement of genetic alterations, abnormal metabolism as well as immunity [4]. However, the precise mechanisms underlying these events remain unclear.

Cancer initiation and progression could be attributed to the accumulation of genetic alterations, which leads to the activation of cellular oncogenes and the inactivation of tumor suppressor genes [5, 6]. Besides, abnormal metabolisms, which were common in cancer cells, were also stated to involve in tumorigenesis [7]. Generally, the metabolism of neoplastic cells were subjected to reprogram in order to support the proliferation, invasion as well as migration. Take glucose metabolism for example, the cancer cells preferentially converted the glucose to lactate and other substrates in order to support their propagation and progression [8]. Meanwhile, increasing evidences suggested that cancer cells show multiple alterations in lipid metabolism, which could affect cell structure, homeostasis and signaling transduction. These abnormal change alone or in combination occasionally affect numerous cellular processes, including cell growth, proliferation, differentiation and motility [9, 10], and was said to be pro-tumoral in various tumors.

PLD1 is phosphatidylcholine-specific phospholipase, which catalyzes the hydrolysis of phosphatidylcholine in order to yield phosphatidic acid and choline. The enzyme may play a role in signal transduction and subcellular trafficking. Alternative splicing results in multiple transcript variants with both catalytic and regulatory properties. As reported, PLD1 was overexpression in most human malignancies associating with malignant phenotypes [11]. For instance, prior studies indicated that PLD1 was upregulated in cancers of the intestinal [12] and breast [13]. Functionality analysis showed that PLD1 overexpression was positively correlated with angiogenesis, invasion and distant metastasis as well as chemoresistence [14, 15]. As to glioma, shattered reports showed that PLD1 and its isoform PLD2 were upregulated in cell lines of glioma associating with increased capacities of invasion and migration [16, 17]. Despite of these advancements, little were known about its expression as well as function in the clinics samples.

In the present study, the expression and biological significance of PLD1 were investigated on the clinical samples of glioma as well as the cell lines using immunohistochemistry and western blot. Additionally, we also evaluated the role of PLD1 in glioma proliferation and migration. We showed that PLD1 was overexpression in glioma samples. Meanwhile, we also found that PLD1 expression was positively correlated with differentiation of glioma cells, and it confers a poor prognosis for the patients. Moreover, we showed in the COX regression assay that that PLD1 was a risk factor for glioma. Consistently, we also showed that PLD1 was overexpression in glioma cell lines. Finally, functional analysis showed that PLD1 overexpression could facilitate the propagation of glioma cells in a manner dependent on CyclinD1 as well as CKD4, and promote the migration of glioma by upregulating MMP9 secretion.

## RESULTS

### PLD1 was overexpression in glioma tissues

**Figure 1:**
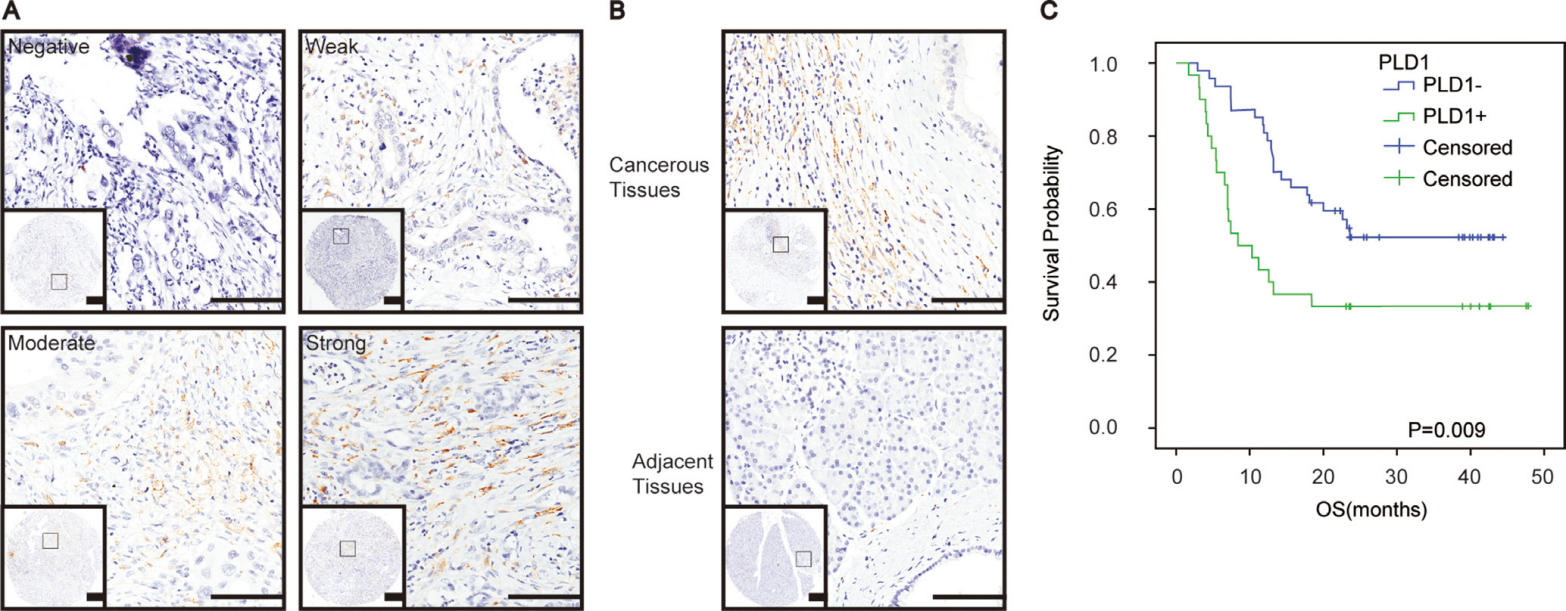
PLD1 was upregulated and responsible for a poor prognosis in glioma (**A**) Representative immunohistochemical staining of PLD1 in glioma, ranging from Negative, Weak, Moderate, Strong positive staining; (**B**) PLD1 expression was more abundance in the cancerous tissues than the paired adjacent tissues in glioma; (**C**) Overall survival curves based on PLD1 expression in glioma. The yellow coloured part that round the cancer cell indicated PLD1 staining, Bar: 100 um.

To examine the biological significance of PLD1 in glioma, its expression was initially detected by immunohistochemistry in the clinical samples. As shown in Figure 1A, PLD1 was cytosolic staining, and it expressed ubiquitously with different levels ranging from negative to strong staining among all the patients. Furthermore, statistical analysis showed that more patients showed positive PLD1 staining (75.2%, Table 1). Also, we found a significant a significant difference of PLD1 expression between the cancerous tissues and the paired none cancerous tissues (*p <* 0.05, Figure 1B, Table 1). Taken together, these data indicated that PLD1 was overexpression in giloma samples.

**Table 1:** PLD1 expression between the cancerous tissues and the adjacent tissues of glioma

	Number	PLD1		*P*
		Positive	Negative	
**cancerous tissues**	129	97 (75.2%)	32 (24.8%)	*P* < 0.05
**Adjacent tissues**	129	27 (20.9%)	102 (79.1%)	

### PLD1 overexpression associated with poor differentiation and prognosis for glioma patients

Subsequently, correlation assay was conducted to examine the relationship between PLD1 and the clinicopathlogical parameters of the patients. As shown in Table 2, we revealed a positive association between PLD1 overerxpression and poor differentiation (*p* = 0.04) of the patients. However, we did not find any significant correlations between PLD1 expression and other parameters of the patients. Meanwhile, Kaplan–Meier analysis indicated that PLD1 overexpression confers a poor prognosis for the patients (*p* = 0.009, Figure 1C).

**Table 2:** Correlation between PLD1 and clinicopathologic parameters of glioma patients

Clinical parameters		Number	PLD1 expression		*P*
			Positive	Negative	
**Age**	> 60	64	49 (38.0%)	15 (11.6%)	0.721
	≤ 60	65	48 (37.2%)	17 (13.2%)	
**Gender**	Male	93	69 (53.5%)	24 (18.6%)	0.672
	Female	36	28 (21.7%)	8 (6.2%)	
**BMI**	< 18.5	22	16 (12.4%)	6 (4.6%)	0.271
	18.5–24.9	62	47 (36.4%)	15 (11.6%)	
	> 25	24	14 (10.9%)	10 (7.8%)	
**Resection**	Partial	10	7 (5.4%)	3 (2.3%)	0.692
	Total	119	90 (69.8%)	32 (24.8%)	
**Tumor size**	< 5 cm	68	50 (38.8%)	18 (14.0)	0.723
	≥ 5 cm	59	45 (34.9%)	14 (10.9)	
**Nuclear grade**	T1/T2	34	27 (20.9%)	7 (5.4%)	0.507
	T3/T4	95	70 (64.3%)	25 (19.4%)	
**Differentiation**	Well	21	19 (14.7%)	2 (1.6%)	0.004
	Moderate	30	16 (12.4%)	14 (10.9%)	
	Poor	78	62 (48.1%)	16 (12.4%)	
**Necrosis**	Positive	62	48 (37.2%)	14 (10.9%)	0.573
	Negative	67	49 (38.0%)	18 (14.0%)	
**WHO Grade**	I/II	58	44 (34.1%)	14 (10.9%)	0.874
	III/IV	71	53 (41.1%)	18 (14.0%)	

### PLD1 was a risk factor for glioma

Considering the fact that PLD1 overexpression confers a poor prognosis for glioma, we examined the prognostic value of PLD1 in glioma using COX regression model. As shown in Table 3, we observed that PLD1 overexpression was a risk factor for the patients (*p* = 0.018, HR = 0.461, 95% CI = 0.243–0.877). Interestingly, the analysis indicated that WHO grade of glioma functions as an independent prognostic factor for glioma (*p* = 0.01, HR = 2.378, 95% CI = 1.228–4.606).

**Table 3:** Multivariate survival analysis of clinicopathologic parameters of glioma patients

Clinical parameters	HR	95% CI	*P*
**Age**	0.686	0.351–1.339	0.269
**Gender**	0.461	0.202–1.051	0.066
**BMI**	1.45	0.693–3.034	0.324
**Resection**	0.977	0.306–3.120	0.969
**Tumor size**	1.192	0.620–2.292	0.599
**Nuclear grade**	0.496	0.219–1.124	0.093
**Differentiation**	1.023	0.411–1.023	0.961
**Necrosis**	0.948	0.175–5.143	0.95
**WHO grade**	2.378	1.228–4.606	0.01
**PLD1 expression**	0.461	0.243–0.877	0.018

### PLD1 promote the proliferation and invasion of glioma cells

**Figure 2:**
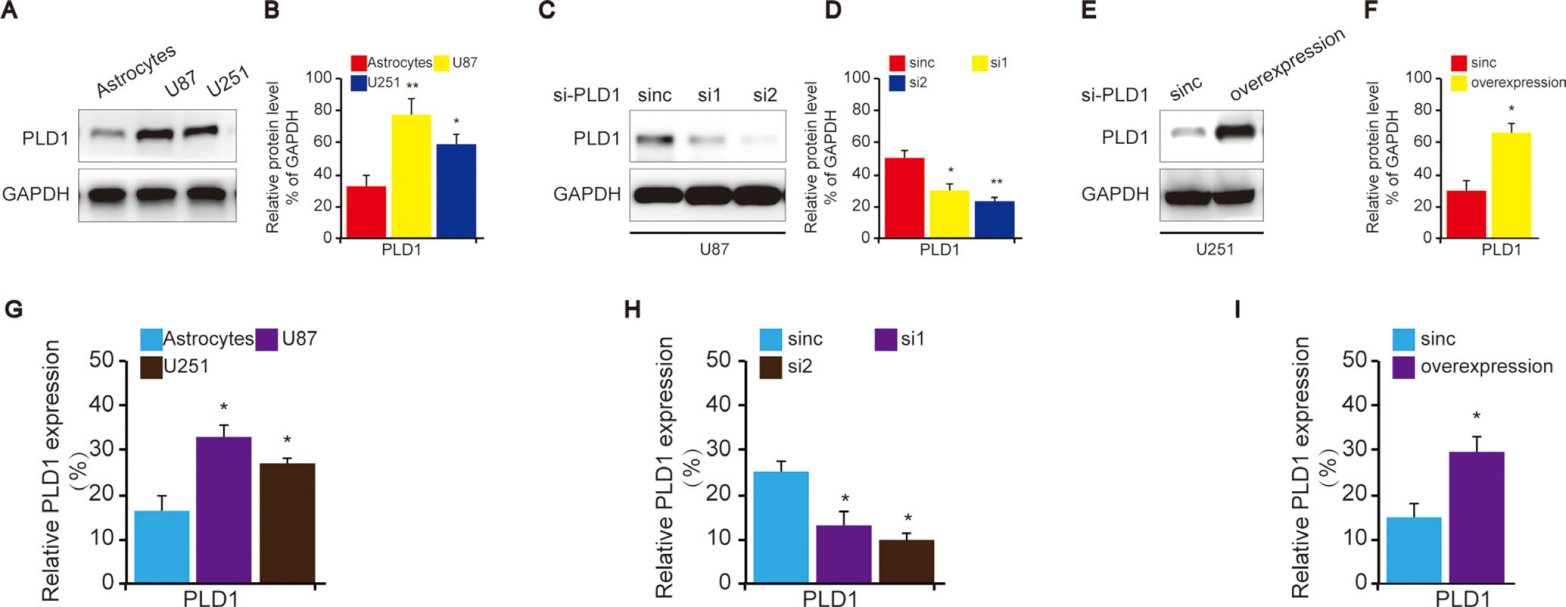
The construction of PLD1 knockdown and overexpression glioma cells (**A**) The expression of PLD1 in glioma cell lines as well as astrocytes. (**B**) Relative PLD1 protein level was quantitated by Image J using GAPDH as an internal control for protein loading. (**C**) The confirmation of PLD1 knockdown in U87 cells using western blot; (**D**) Relative PLD1 protein level was quantitated by Image J using GAPDH as an internal control for protein loading. (**E**) The confirmation of PLD1 overexpression in U251 cells using western blot; (**F**) Relative PLD1 protein level was quantified by Image J using GAPDH as an internal control for protein loading. Relative densities (RD) are presented as mean ± SE. (*n* = 3) of the fold change relative to the internal control. GAPDH was used as an internal control for protein loading. (**G**) The detection of PLD1 expression in the cells of astrocytes, U87 and U251 in RNA levels using qPCR. (**H**) The detection of PLD1 expression in U87 cells U251 in RNA levels upon PLD1 knockdown using qPCR. (**I**) The detection of PLD1 expression in the cells U251 in RNA levels upon PLD1 overexpression using qPCR. Data are expressed as mean±S.E from three independent experiments, *P < 0.05 and **P < 0.01.

To confirm the role of PLD1 in glioma, we then examined its expression and function in cell lines of glioma. To begin with, PLD1 expression was investigated using western blot. The data showed that the neoplastic cells exhibited high levels of PLD1 expression compared to astrocytes (Figure 2A, 2B). In addition, we also revealed that PLD1 was overexpression than astrocytes in RNA levels (Figure 2G).

Subsequently, in order to evaluate the role of PLD1 in glioma, U87 cell was selected to construct PLD1-knockdown cells since it showed higher PLD1 expression compared to U251 (Figure 2A, 2C, 2D). Correspondingly, U251 cell was used to construct PLD1-overexpression cells (Figure 2A, 2E, 2F). The effects of the construction were also confirmed by q-PCR (Figure 2H, 2I)

**Figure 3:**
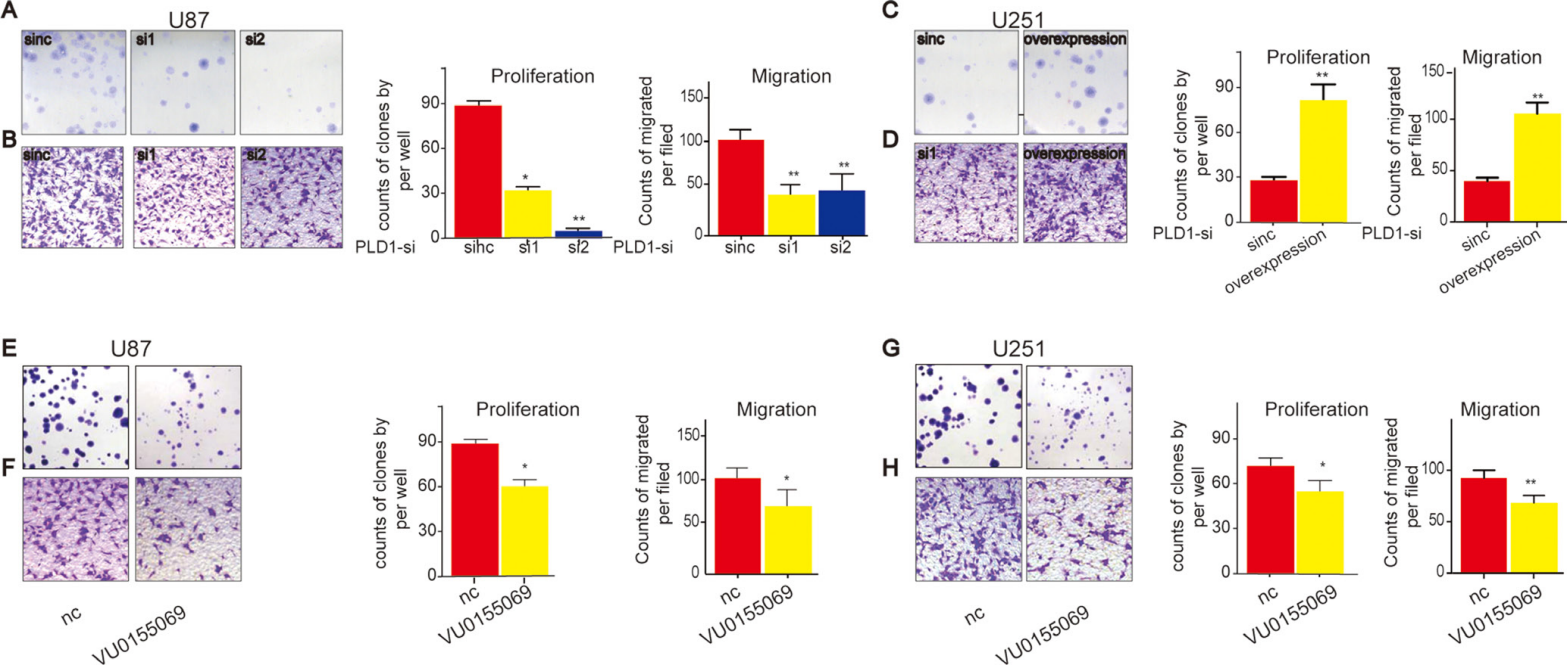
PLD1 overexpression facilitate proliferation and migration of glioma cells (**A**–**B**) U87 cells showed decreased proliferation (A) and migration (B) upon PLD1 knockdown; (**C**–**D**) U251 cells showed increased proliferation (C) and migration (D) upon PLD1 overexpression; (**E**–**F**) U87 cells showed decreased proliferation (E) and migration (F) upon PLD1 inhibition; (**G**–**H**) U251 cells showed decreased proliferation (G) and migration (H) upon PLD1 inhibition. 48 hours after transfection, the cells were subjected to experimental detection, such as PLD1 expression and the proliferation and migration. The PLD1-selective inhibitor (VU0155069) with the concentration 1 um was used to affect the cells. 72 hour later, the migration and proliferation of glioma cells were examined. Data are expressed as mean ± S.E from three independent experiments, with significant differences from control designated as*P < 0.05. **P < 0.01.

After that, we evaluated the capacities of proliferation and migration of the treated cells. We found that U87 cells exhibited decreased proliferation (Figure 3A) and migration. (Figure 3B) upon PLD1 knockdown. Consistently, we revealed that U251 cells showed elevated proliferation (Figure 3C) as well as migration (Figure 3D) upon PLD1 overexpression. To further confirm the role of PLD1 in glioma proliferation and migration, we pharmacologically inhhibited PLD1, and found that both U87 and U251 cells cells exhibited decreased proliferation (Figure 3E, 3G) and migration. (Figure 3F, 3H) upon PLD1 inhibition.

**Figure 4:**
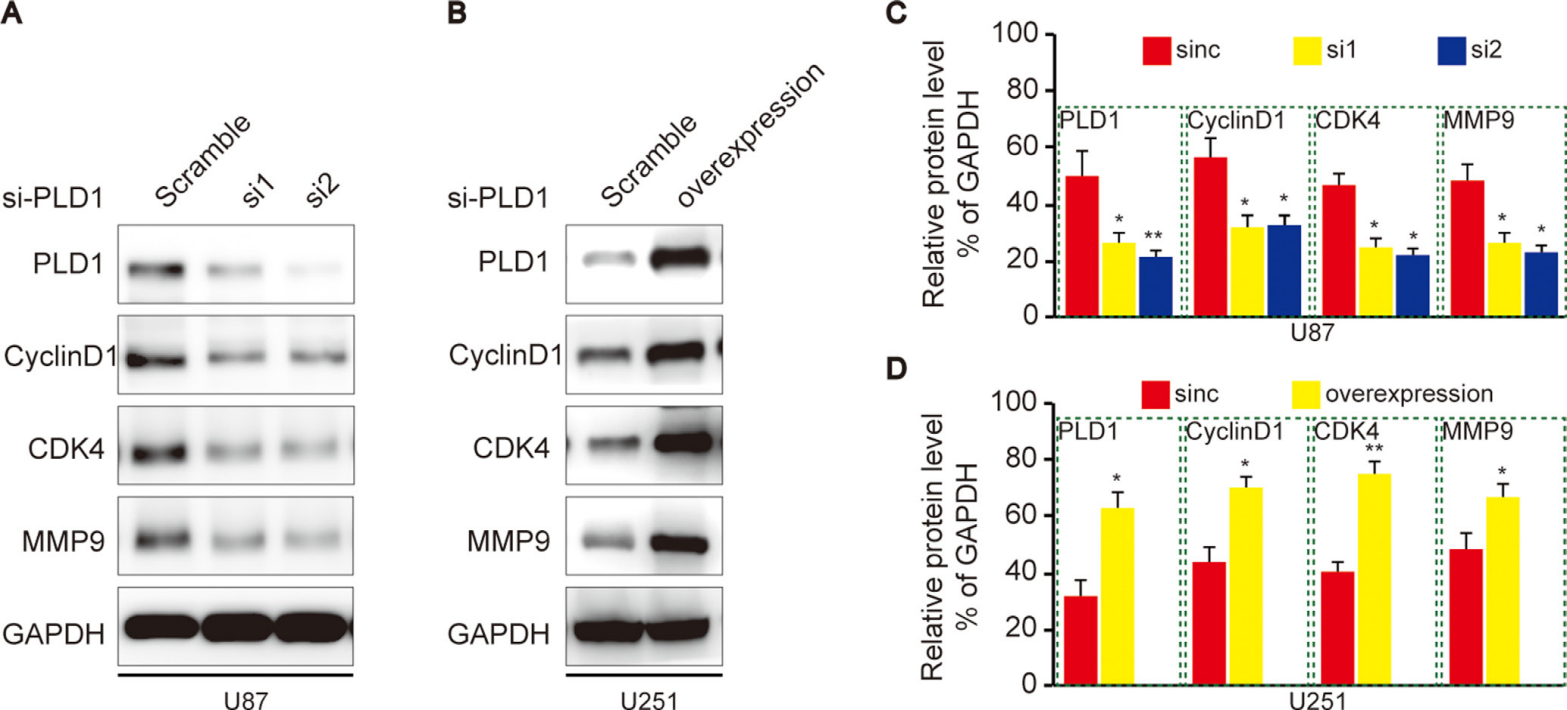
PLD1 regulates CyclinD1, CDK4 and MMP9 expression in glioma cells (**A**) U87 cells showed decreased CyclinD1, CDK4 and MMP9 expression upon PLD1 knockdown; (**B**) Relative PLD1, CyclinD1, CDK4 and MMP9 protein level were quantitated by Image J using GAPDH as an internal control; (**C**) U251 cells showed increased CyclinD1, CDK4 and MMP9 upon PLD1 overexpression; (**D**) Relative PLD1, CyclinD1, CDK4 and MMP9 protein level were quantitated by Image J using GAPDH as an internal control. Relative densities (RD) are presented as mean 6 S.E. (*n* = 3) of the fold change relative to the internal control. GAPDH was used as an internal control for protein loading. Data are expressed as mean±S.E from three independent experiments, *P < 0.05 and **P < 0.01.

Since CyclinD1 and CDK4 facilitate cell proliferation in various cells, we then investigated whether they involved in PLD1-resultant proliferation of glioma cells. To this end, we examined their expression upon PLD1 knockdown in U87 cells, and found that they both decreased correspondingly (Figure 4A, 4C). On the contrary, U251 cells showed elevated CyclinD1 and CDK4 expression upon PLD1 overexpression (Figure 4B, 4D). Meanwhile, since MMP9 faciliates migration of various cancers, we then examined whether it also implicated in PLD1-resultant migration of glioma cells. Using western blot assay, we showed that MMP9 expression decreased upon PLD1 knockdown (Figure 4A, 4C). Correspondingly, U251 cells exhibited elevated MMP9 expression upon PLD1 overexpression (Figure 4B, 4D). Overall, these data suggested that PLD1 overexpression was a risk factor for the migration and proliferation of glioma.

## DISCUSSION

Recently, mounting studies had studied the role of lipids metabolism in the initiation and progression of human malignancies, and most of them indicated that PLD1 was pro-tumoral in these malignancies [9]. In the present study, we learned the role of lipid metabolism in glioma on the cell line and tissues by studying PLD1, a critical enzyme that implicated in lipids metabolism. We found that upregulated PLD1 was assiciated with poor differentiation and prognosis for glioma. Meanwhile, we also showed that PLD1 overexpression could facilitate the proliferation and migration of glioma cells. Overall, our data proposed that PLD1 overexpression was pro-tumoral in glioma, and that targeted inhibition PLD1 might represent a novel approach for glioma management.

Previously, multiple studies had examined the role of PLD1 in human diseases, including cancers [9, 18]. Of note, most of the studies concluded that PLD1 overexpression was pro-tumoral [11, 19], and that PLD1 was a potential therapeutic target for human cancers. Of note, there were also studies focused on the role of PLD1 in glioma, they put forward that PLD1 was overexpression in glioma cell lines, and that PLD1 was responsible for aggressive phenotype [20]. Similarly, our study confirmed the positive role of PLD1 in glioma development from both the cellular and histological levels. Additionally, we also confirmed that PLD1 overexpression promotes proliferation as well as migration in a manner dependent on CyclinD1, CDK4 and MMP9 respectively.

Indeed, insights into PLD1 promoted more and more researchers to learn whether the inhibition of PLD1 was an alternative approach for cancer treatment. Based on the assumption, various studies had started in the special field [21]. For instance, Kang DW and colleagues showed that rebamipide, a mucosal-protective antiulcer agent, contributes to anti-tumorigenic effect of gastric cancer cells via the inhibition of the H. pylori cagA-NF-κB-PLD1 signaling pathway [22]. Meanwhile, some other reports suggested that PLD1 inhibition in combine with ionizing radiation was effective in inducing DNA damage, and breast cancer cell apoptosis subsequently [23]. As to glioma, Park MH and colleague revealed that quercetin could inhibit the invasion and proliferation of glioma cells via abolishes PLD1 [17]. This verr in combine with our data suggedted that targeted inhibition of PLD1 might represent a novel direction for the management of glioma.

However, it should be noted that there were limitations of our study. First, as shown in Table 2, some important data, such as BMI and tumor size of some patients were missing, which might subsequently lead to a misunderstanding on the correlation between PLD1 and BMI/tumor size. In addition, we proposed the conclusion in our study that PLD1 inhibition might be an alternative approach for the management of glioma based solely on the data *in vitro* without any data on *vivo*. Furthermore, our study did not give the detailed mechanisms whereby PLD1 interacts with CyclinD1, CDK4 and MMP9. Obviously, these data was not solid enough for the conclusion.

In summary, our findings had revealed that PLD1 overexpression confers a poor prognosis for patients with glioma. In addition, we further showed that PLD1 could facilitate the proliferation and migration of glioma cell lines. Since PLD1 serves as a pro-tumoral factor for glioma, further studies are needed to determine the pathological mechanism of PLD1 in glioma so as to reveal novel therapeutic targets for the lethal disease.

## MATERIALS AND METHODS

### Patients

129 patients with histopathologic diagnosis of glioma (ICD, Tenth Revision, codes C25) were included in our study. Glioma cancer tissues and paired adjacent normal tissues were collected from the department of pathology, tertiary hospital in hangzhou area from 2012 to 2014. The last follow-up was on June 28th, 2016. The patients’ clinicopathlogical characteristics include age, gender, WHO stage and so on. Each patient provided written informed consent and the study was approved by Ethics Committees tertiary hospital in hangzhou area.

### Tissue microarray construction

The microarray was made as described [24]. Briefly, H&E-stained sections were made from primary tumor blocks to define tumor regions. Representative tumor regions are defined as areas with at least 75% cancer cells without necrosis. Tissue cylinders (1.5 mm in diameter) were then punched from the regions of the block using a tissue microarrayer (Gentury, IL, USA) and placed into recipient paraffin blocks. Sections of the TMA blocks were transferred to glass slides.

### Immunohistochemistry (IHC)

In brief, the tissue microarrays were dewaxed and dehydrated in xylene and alcohol bath solutions, respectively. Endogenous peroxidase activity was blocked using 0.3% hydrogen peroxide for 10 mins, before antigen retrieval was undertaken by setting the slides in 0.01 M citrate buffer (pH 6.0) at 98°C for 5 mins using a microwave oven. The slides were cooled to room temperature and blocked by incubating with normal goat serum at room temperature for 1h, followed by incubation at 4°C overnight with the primary antibodies (Cell Signaling Technology, Beverly, MA, USA). Finally, the sections were incubated with HRP-labeled secondary antibody and visualized using diaminobenzidine. Specifically, the dilution of the primary antibody used in the experiment was 1:100.

### Evaluation of IHC

Evaluation of the staining was performed by two independent pathologists blind to the research in at five areas at 400× magnification. The staining was scored according to the intensity and percentage of the stained cells. Staining intensity was assigned as 0 (No staining), 1 (weak staining), 2 (Moderate staining), and 3 (Strong staining). The percentages were classified into: 1 (≤ 25%), 2 (25%–50%), 3 (50%–75%), and 4 (75%–100%). The final score of staining was calculated as the staining intensity × the percentage of stained area. For statistical analysi, a score < 6 was regarded as negative expression, and > 6 as positive expression.

### Cell lines and cell culture

The glioma cell lines U87, U251 and normal astrocytes were purchased from Hangzhou for the coreof Science biological technology co., LTD. All the cells were cultured and grown in RPMI 1640 (Gibco, BRL, CA, USA) supplemented with 10% fetal bovine serum (FBS, Gibco, Carlsbad, CA, USA) at 37°C in a humidified atmosphere of 95% air and 5% CO2. Cells with gene deletion/overexpression were cultured in the same condition with 1.5-μg/mL puromycin (Sigma-Aldrich, St. Louis, MO, USA).

### Transient transfection

This procedure was conducted to construct PLD1 knockdown and overexpression cells. Glioma cells were cultured at 37°C in RPMI 1640 (Gibco, BRL, CA, USA) containing 10% FBS and 1% antibiotic–antimitotic. Cells were grown to 50–60% confluence for transient transfections using Lipofectamine Plus (Invitrogen, CA, USA) according to the manufacturer’s instructions. The The sequences for PLD1 knockdown and overexpression was borrowed from the study by Rocky Cipriano [25].

### Western blot analysis

Cells were washed three times with cold PBS and lysed on ice in RIPA buffer with protease inhibitors PMSF (Beyotime Biotechnology, China). Protein concentrations were determined by BCA method (Beyotime Biotechnology, China). A total of 20 μg protein was separated by 10% SDS-PAGE and electro-blotted onto NC membranes using semi-dry blotting apparatus. After blocking in 3% bovine serum albumin (FBS, Gibco, Carlsbad, CA, USA), the membranes were incubated with the primary antibodies overnight at 4°C. The membranes were washed and incubated with the secondary antibodies for 1 h at room temperature on a shaker. The protein bands were visualized using a commercially available enhanced chemiluminesence kit (Thermo Scientific, Hudson, NH, USA). GAPDH were used as control. The primary antibodies used in the study include: PLD1 (Cat No, 3832, CST, Beverly, MA, USA), CyclinD1(Cat No, 2978, CST, Beverly, MA, USA), CDK4 (Cat No, 12790, CST, Beverly, MA, USA) and MMP9 (Cat No, 13367, CST, Beverly, MA, USA); and GAPDH (Santa Cruz Biotechnology, CA, USA). The bands were were quantified using Image J as described previously [26]. The dilution for the antibody used were listed: CyclinD1, CDK4 and MMP9, 1:1000; GAPDH, 1:5000.

### Colony formation assay

Briefly, ∼1,000 cells were added to each well of a 6-well culture plate. After 2 weeks incubation, cell colonies were washed twice with PBS, fixed with 4% paraformaldehyde for 15 mins, and then stained with crystal violet for 30 min. Individual clones with > 50 cells were counted.

### Cell migration assay

For migration assays, conditioned media without FBS were added to the upper chambers of 24-well tissue culture plates in triplicate. Glioma cells (40,000) were added to the upper chambers of Transwell assays (BD Biosciences, Franklin Lakes, NJ, USA). Complete medial was added to the bottom champer. Cells were allowed to migrate for 14 h and then fixed, stained, and quantified.

### Statistical analysis

Statistical analysis were performed using SPSS software (version 21.0; SPSS Inc, Chicago, IL, USA). The relationships between the clinicopathlogical factors and PLD1 expression were investigated using Pearson χ^2^ test and Spearman’s rank test. Kaplan–Meier analysis was used to demonstrate PLD1 in the overall survival (OS) of glioma. Cox regression model was used to evaluate the prognostic value of each factor. Results were considered statistically significant when *p* < 0.05.
